# “Are we working (too) comfortably?”: a focus group study to understand sedentary behaviour when working at home and identify intervention strategies

**DOI:** 10.1186/s12889-024-18892-1

**Published:** 2024-06-06

**Authors:** Sarah Morton, Claire Fitzsimons, Divya Sivaramakrishnan, Ruth Jepson, Ailsa Niven

**Affiliations:** 1 Physical Activity for Health Research Centre, University of Edinburgh, Edinburgh, EH8 8AQ UK; 2 Scottish Collaboration for Public Health Research and Policy, University of Edinburgh, Edinburgh, EH8 8AQ UK

**Keywords:** COM-B, Sedentary, Sitting, Occupational, Work, Teleworking, Home-working, Health

## Abstract

**Background:**

The Covid-19 pandemic initiated an enduring shift in working patterns, with many employees now working at home (w@h). This shift has exacerbated existing high levels of occupational sedentary behaviour (SB) in office workers, which is a recognised risk to health and well-being. This study aimed to use the Capability-Opportunity-Motivation-Behaviour (COM-B) model to better understand both employees’ SB, and line managers behaviour to assist employees to reduce SB when w@h, and identify how employees can best be supported to reduce SB.

**Methods:**

Three online focus groups with employees aged 18–40 working in desk-based roles (e.g. administrative / sales / customer services) (*n* = 21), and three with line managers (*n* = 21) were conducted. The focus groups facilitated discussion regarding participants’ current behaviour, what impacts it, and what could be done to reduce employee SB when w@h. The focus group data were thematically analysed guided by the COM-B framework to understand influences on behaviour, and to identify promising intervention strategies.

**Results:**

Most participants recognised that w@h had elevated employee occupational SB, and line managers reported the importance of supporting employees to manage their workload, and encouraging and modelling taking breaks. There were multiple influences on both employee and line manager behaviour with capability, opportunity and motivation all perceived as influential, although not equally. For example, a major theme related to the reduced physical opportunities for employees to reduce their SB when w@h, including blurred work-life boundaries. Changes in physical opportunities also made supporting employees challenging for line managers. Additionally, the w@h environment included unique social opportunities that negatively impacted the behaviour of both groups, including an expectation to always be present online, and social norms. A range of strategies for reducing SB when w@h at both individual and organisational level were suggested.

**Conclusions:**

It was evident that SB when w@h is influenced by a range of factors, and therefore multi-component intervention strategies are likely to be most effective in reducing SB. Future intervention research is a priority to evaluate and refine strategies, and inform w@h guidance to protect both the short-term and long-term health consequences of elevated SB for those who continue to w@h.

**Supplementary Information:**

The online version contains supplementary material available at 10.1186/s12889-024-18892-1.

## Background

Sedentary behaviour (SB) is defined as any waking behaviour in a sitting, lying or reclining posture with low energy expenditure (≤ 1.5 METs) [1]. SB is a public health hazard with higher levels associated with adverse physical and mental health consequences [2–5]. Health guidelines recommend adults minimise time spent sedentary, and when possible break up periods of inactivity with at least light physical activity [6].

SB is prolific across modern lifestyles and can take place in a range of contexts [7]. The occupational context is a high risk setting for SB as illustrated by sedentary time accounting for 81.8% of work hours in office workers [8]. The occupational context changed dramatically with the Covid-19 pandemic when lockdown restrictions required many workers to shift from working in an office to working at home (w@h) [9]. This shift to w@h resulted in further exacerbation of high levels of occupational SB [10, 11]. This exacerbation is concerning because the trend for w@h remains, with recent UK data indicating that the percentage of adults who had worked at home at some point in the last seven days had increased from ∼ 12% pre-Covid to ∼ 40% in 2023 [12].

Interventions are needed to support employees to reduce and break up SB when w@h. The Medical Research Council (MRC) framework [13] provides guidance on developing and evaluating complex interventions, and an initial step for developing an intervention is to consider if an existing intervention can be adapted to a new context. To this end, we conducted a rapid evidence review of what works to reduce SB in an office setting [14], and with stakeholder input, appraised if effective strategies could be transferred to w@h. The most promising intervention strategies we identified were educational materials, use of role models, incentives and prompts. Intervention research specifically on the topic of SB while w@h has been limited to date, with one US pilot study successfully intervening to reduce SB in the short-term by providing height-adjustable desks, or an online SB modification programme, or combination [15, 16].

These studies contribute to the growing evidence base in this area, however, in line with intervention development frameworks, there is also a need to better understand the target behaviour to inform future intervention design [13, 17]. Within the Behaviour Change Wheel (BCW), the COM-B model provide a theoretical structure to guide this process [17]. The COM-B model articulates that Behaviour is influenced by Capability, Opportunity and Motivation. Capability relates to having the physical (e.g., stamina) and psychological (e.g., knowledge) capability to perform the behaviour. Opportunity relates to external factors that make the behaviour possible and includes physical (e.g., having adequate space) and social (e.g., supportive colleagues) opportunities. Capability and opportunity can directly influence behaviour, and indirectly via impacting motivation. Motivation to engage in the behaviour refers to the internal processes that influence behaviour and can be sub-divided into reflective (i.e., involving planning and evaluation) and automatic (e.g., emotions) motivation.

In a study with university staff w@h [11], we previously used a COM-B specific questionnaire [18] and open-ended questions to better understand SB when w@h. Findings highlighted key influences on behaviour (e.g., perceived low levels of social opportunity), and from these findings potential intervention strategies were identified (e.g., implement social support and role modelling). However, a questionnaire can be limited in terms of the depth and richness of information collected, and lack of opportunity for further elaboration. Alternative methods such as interactive focus groups would facilitate greater understanding. Additionally given that role modelling and social support were identified as promising intervention strategies, there is important value in also understanding managers’ perspectives on how they can best support employees to reduce and break up SB. Line managers are those who are directly responsible for employees, and are therefore likely to have most influence on employees’ behaviour. To date, no research has considered line managers’ perspectives on the issue of reducing SB in the w@h environment.

Although it is likely that all employees w@h could benefit from reducing SB, the greatest public health gain will be in those individuals whose health will benefit in both the short and longer term. Adults aged 18–40 years may work for much of their career in a working from home or hybrid environment, and due to levels of workplace SB this age group has already been identified as high risk for SB [19]. Job type can also influence occupational SB with those in typically desk-based roles (e.g., administrative/secretarial, sales and customer services) demonstrating some of the highest levels of SB [20], and therefore have the potential for greatest benefit by reducing their SB.

This study aimed to use a series of focus groups guided by the COM-B model to consult with both employees aged 18–40 in non-managerial desk-based roles, and line managers to better understand the target behaviour of SB when w@h and understand what needs to change to support reduced SB. To address this aim there were five RQs:

1. How do employees describe their SB when w@h.
2. What do employees perceive influences their ability to reduce/break up SB when w@h?
3. How do line managers describe their behaviour to support employees to reduce SB when w@h?
4. What do line managers perceive influences their ability to support employees to reduce SB when w@h?
5. What are the strategies that employees and line managers think may help employees to reduce their SB when w@h?

## Method

This study is written and reported in line with the Consolidated Criteria for Reporting Qualitative Studies (COREQ)(Supplementary file 1).

### Participants and recruitment

Employees and line managers were purposively recruited to this study using multiple pathways to meet inclusion criteria (see Supplementary File 2) (e.g., social media via Twitter) between December 2021 and February 2022 with the aim of recruiting up to 24 employees and 24 line managers for eight participants in each of six focus groups. The target sample size was based on pragmatic considerations (i.e., time, managing focus group interactions), and the principles of information power [21]. Inclusion criteria for employees included working full-time in a mostly desk-based role (e.g. administrative, clerical, customer service sector) with no line management responsibilities, aged 18–40, and working at home for more than 50% of their working week. Inclusion criteria for line managers included being directly responsible for individuals who were mostly working in desk-based roles, working full time, and spending more than 50% of their working week at home. Participants from the employee group were offered a £30 gift voucher as a token of appreciation for participating in an activity that extended beyond their role responsibilities. Potential participants who expressed interest in the study were sent further information. Participants who met the inclusion criteria and completed the online consent were invited to participate. Supplementary file 2 illustrates the participant flow from recruitment to completed focus group.

### Focus groups

Three employee and three line manager focus groups (60 min each) were facilitated and recorded (20 January 2022–24 February 2022) on MS Teams by at least three members of the research team (AN, SM, CF, DS). All members of the research team were experienced qualitative researchers with PhDs. Pre-determined core questions guided by the COM-B model were presented via PowerPoint in three stages. The core questions were supplemented by prompts and probes, where appropriate and ice-breaking questions were included at the beginning of each focus group. The researchers intentionally sought to include all participants in the discussion, where appropriate. A preliminary focus group with researchers w@h was used to trial and refine the format ahead of the focus groups proper (Example focus group materials available as Supplementary File 3).

The first stage of the focus group collected information about participants’ knowledge and awareness of SB and health consequences. Recently, it has been noted that the sedentary behaviour of sitting is an ‘invisible’ behaviour [22], so we took steps to raise participants’ awareness of the behaviour. Specifically, in advance of the focus group both groups were sent an infographic (see Supplementary File 4), designed by the researchers, outlining background information about SB, and how this had changed due to an increase in w@h. Additionally, the researchers presented brief information on the topic to further stimulate discussion. The second stage of the focus group considered current SB when w@h and what influences this behaviour. For the employees, this question focused on their own behaviour, and the line managers were asked to consider the behaviour of their employees, and their own supportive behaviour. The third stage focused on what could be done to reduce long periods of SB when w@h. Employees were asked to consider how they could reduce their own SB, and line managers were asked how they could support their employees to reduce their SB. Participants were also invited to comment on strategies identified from a rapid review of what works to reduce SB in offices and may transfer to a w@h context [14]. After each focus group, the research team met to debrief on main findings and consider any improvements or changes to make to the subsequent focus groups. The focus groups were recorded using the MS Teams meeting recording function. Recordings were professionally transcribed verbatim, and on return checked for completeness and accuracy, and any identifiable details were removed and participant names replaced with pseudonyms.

### Analysis

The data were thematically analysed within an interpretive paradigm and drawing from the principles of thematic analysis [23]. Transcripts were reviewed and data representing a relevant point made by participants were extracted by one researcher (SM) initially into NVivo 20, and then in MSWord documents. For both employee and line manager data, extracted text were initially deductively organised into broad categories guided by COM-B relating to capability (physical and psychological), opportunity (social and physical), motivation (automatic and reflective) and behaviour by one researcher (SM). Within these broad categories, sub-themes were identified by inductively clustering together text with similar meaning, with sub-themes incorporated, where appropriate. The organisation of the data into COM-B categories and the identification of the sub-themes was discussed, refined, and agreed by at least two researchers to encourage reflection and discussion. Matrices were then used to create tables illustrating the COM-B categories and themes, with further refinements made at this point to enhance the coherence between and within themes. Final themes were discussed and agreed on by all researchers. Participants’ suggestions for reducing SB while w@h were extracted from the transcripts and inductively clustered into broad themes. Additionally, responses to the strategies identified from the rapid review [14] were considered to determine whether the participants viewed they would be feasible in the w@h environment.

## Results

Table 1 shows participant characteristics. Although the focus was on reducing SB, comments relating to all movement behaviours (e.g., physical activity, exercise) were included as they may have a role in reducing SB. Throughout relevant comments are included, even if reported by only one participant. We deliberately avoided quantifying the qualitative data, however for transparency we indicated where only one or two participants highlighted the point.

**Table 1:** Demographic characteristics of employee and line manager focus group participants

Characteristic	Employees (*n* = 21)	Line managers (*n* = 21)
Age, *n* (%)		
18–25	-	-
26–30	9(42.9%)	-
31–35	4(19%)	1(4.7%)
36–40	6(28.6%)	2(9.5%)
41–45	1(4.7%)	2(9.5%)
46–50	-	6(28.6%)
51–55	-	7(33.3%)
61–65	-	1(4.7%)
Missing	1(4.7%)	2(9.5%)
**Gender, n (%)**		
Female	16(76.2%)	16(76.2%)
Male	5(23.8%)	3(14.3%)
Missing	-	2(9.5%)
**Ethnicity, n (%)**		
White	12(57.1%)	13(57%)
Black American	1(4.7%)	-
Black British	1(4.7%)	-
Scottish Pakistani	1(4.7%)	
Missing	6(28.6%)	8(29%)

### Employees

#### Employee perceived characteristics of SB while w@h

The majority of participants commented that their SB was elevated whilst w@h compared to when working in the office, primarily due to sitting more but also because they were not moving much during the working day. Some participants also reported their sitting was ergonomically ‘poor’. A small number of participants indicated they had strategies to reduce SB, for example moving during the day. Finally, only one participant explicitly stated that their sitting time had not changed.

#### Influences on employee’s behaviour to reduce and/or break up SB when w@h

##### Employee physical and psychological capability

As illustrated in Table 2, there were limited comments about physical capability, and participants acknowledged that having the physical capability to reduce SB varied between individuals. For example, it was specifically highlighted that some people cannot stand for long periods. Having physical skills to reduce SB was also noted as beneficial, for example one participant described being able to implement their yoga skills to move more frequently.

**Table 2:** Themes relating to employee capability to reduce SB when W@H

Category	General theme (and impact on reducing SB)	Sub-theme (where evident)
Physical capability	Physical capability varies between individuals (+/-)	-
	Having physical skills (+)	-
Psychological capability	Knowledge of SB (+/-)	Knowledge (or lack) of the SB ‘issue’
		Knowledge (or lack) of elevated SB when w@h
	Have mental energy (+)	-
	Self-monitoring (+)	-
	Using behavioural regulation skills (plan)(+)	-

The category psychological capability included themes about knowledge of SB, having mental energy, self-monitoring, and using behavioural regulation skills to implement opportunities (Table 2). The largest theme related to knowledge about SB, which included two sub-themes. Firstly, a sub-theme about having knowledge (or lack of) about the ‘issue’ of SB was identified as impacting behaviour. Many participants were well informed, and these participants typically had some experience in a health-related area (e.g., Facilities Officer, Health Promotion Officer, Public Health and Wellbeing Officer). Many participants were knowledgeable about health consequences of prolonged SB, with one participant stating, “*we all know the health thing”* (Rosie, 45, Marketing Executive). For other participants, they felt they knew some of the information, but were also surprised and interested to learn that, for example, some cancers and mental health issues can be associated with SB. For some, lack of knowledge was evident as the distinction between PA and SB was unclear, with several participants equating reducing SB with being more active through exercise. For example, one participant stated, “*I do think people have this perception that if they exercise it doesn’t matter that I sat all day”* (Isla, 34, Research Fellow). Another participant reported that the discussion had enhanced their knowledge and clarified in their mind “…*the point about moving throughout the day*” (Oliver, 36, Lecturer) to reduce sedentary time. Notably, one participant highlighted that those not working in health might not be aware of the impact of sitting and associated consequences. Finally, one participant reported they had not known about the concepts of standing and walking meetings, which can be used to break up SB.

A second sub-theme identified related to having knowledge (or lack) of how w@h could contribute to elevated levels of SB, and this related to both individual’s own behaviour and to other workers generally. Specifically, some participants recognised they were sitting much more when w@h, and agreed with the information provided in the infographic about volume of time spent sitting while w@h. For example, one participant noted “*I’m very much aware that working from home probably has increased a lot of people’s sedentary behaviour, and things like that*” (Noah, 26, Research Fellow). However, this knowledge was not universal, and another participant indicated they were not sure if their sitting was more at home or in the office. Further, some participants felt their sitting behaviour was like-for-like in comparison to the office.

A second theme related to having mental energy to reduce SB, identified by two participants as being important, and that sometimes they did not have that drive because of mental health challenges. Additionally, some participants commented on self-monitoring their SB and one noted that it was difficult to do “…’*cause I’m quite busy with work, I can focus more and forget time effectively and end up sitting much longer*” (Freya, 32, Volunteer Co-ordinator). Finally, using behavioural regulation skills to implement intentions, plans and opportunities to be active and reduce SB were mentioned by several participants, particularly arranging to meet others, and identifying and planning when to move during the day.

##### Employee physical and social opportunities to reduce SB

Several themes were identified about physical opportunities afforded (or not) by the w@h environment to reduce SB (see Table 3). The largest theme included participants’ comments about how switching to the w@h environment reduced opportunities to move. A sub-theme was identified relating to loss of movement opportunities between worksites and nearby, and around buildings. For example, Lucy reported “*I definitely sit a lot more when I’m at home and a lot of that’s because of the small movements that you do in the office so like walking to get a coffee or going outside for lunch or walking to meetings and stuff, so that’s definitely had an impact*” (Lucy, 30, Public Health Intelligence Advisor).

**Table 3:** Themes relating to employee opportunities to reduce SB when W@H

Category	General theme (and impact on reducing SB)	Sub-theme (where evident)
**Physical Opportunities**	Reduced opportunity to move (-)	Loss of movement between sites/ nearby/ around buildings
		Loss of commute
		Change in job demands – including Increase in online meetings/ back to back meetings
		Blurred lines between work and home/ extension in the working day
		Small home environment
	Increased opportunity to move (+)	Increased flexibility/longer breaks
		Use lunchtime/ commute time to move
		Eat more regularly
		Plan moving meetings
		Standing during online meetings
	Access to equipment (+/-)	Equipment available to help reduce SB
		Lack of equipment
	Work demands (-)	Desk based job
		Immersive work
		Deadlines/ Pressure
	Influence of season (+/-)	-
	Organisational initiatives (+)	Activities for increased movement
		Flexible work patterns
**Social opportunities**	Social norms around online work (-)	Expectation that attendees will be at their desk
		Camera on indicates ‘presence’
		Can move when camera is off in certain circumstances
	Colleagues encourage activity (+)	Colleagues encourage activity
	Organisational support (+/-)	Supportive/ recognize issue
		Lack of knowledge of how to support
	Others in household (+/-)	Others encourage movement
		Have negative impact

Several participants highlighted that because they no longer had to actively commute into work, they had lost an opportunity to move. For example: “*I never thought I’d say I miss the commute…like, I live in London at the moment, I used to spend an hour commuting, but I miss the walk to the bus, the walk to the…you know, the tube as well”* (Amelia, 30, Public Involvement Co-ordinator). For some, demands of their job had changed significantly from a role involving moving during the day to a more sedentary desk-based role. Many participants highlighted the increase in online meetings, and ‘back-to-back’ meetings being scheduled, resulted in reduced opportunity to move. For several participants, w@h resulted in a ‘blurring’ of lines between work and home life, leading to working extended hours, and therefore spending longer at their desk without moving. One participant commented: “*it’s just so easy to work over your allotted time because you’re right there, you know, there’s not that distinct cut-off point where you’re having your commute to go back home.”* (Luca, 31, Administrator). Several participants reported that their home environment was small and did not provide space to move. For example, Mia reported “*I don’t have a space in my house to work so I work on the kitchen table, so pretty much when it comes to getting lunch, I just have to stand and walk over to the fridge which is only about ten steps away and then come and sit down*” (Mia, 38, GP).

For some participants, it was evident that w@h increased opportunities to move. Participants appreciated the flexibility that w@h offered, allowing longer breaks and active lunchtimes. Some participants implemented their own opportunities to reduce SB by arranging walking meetings, or standing and moving during online meetings. Access to relevant equipment impacted on reducing SB. For example, having a standing desk was perceived as beneficial, whereas not having access to equipment was detrimental. Some participants noted their work demands increased their SB. For example, if their job specifically required them to be at a desk, work was immersive, or they had deadlines then that would increase SB. Participants noted that the seasons impacted on SB, with winter months and dark nights reducing the likelihood they would break up SB and go outside, or leave home to be active at night. In contrast, it was commented that it was easier to be more active in the summer. Several participants reported that their organisations were implementing initiatives to support reducing SB and increase movement (e.g. step count challenges). Additionally, flexible working arrangements were identified as beneficial to enable employees to manage their time and find opportunities to be active during the day. For example, Harper commented “*work is really pushing hybrid and flexible working so they say if you want to work compressed hours that’s fine, if you want to take two hours out to do a gym class, that’s all good, and they’re really, really encouraging that, so I’m taking time out of my day to do online classes and sometimes go for a swim*”. (Harper, 37, Facilities Officer).

Several themes were identified relating to social opportunities afforded (or not) by the w@h environment to reduce SB. Participants discussed social norms around online meetings that impacted on them being able to reduce SB. Specifically, many employees reported that they perceived an expectation to be seated at their desk during meetings, and that having the laptop camera on indicated they were ‘present’. A couple of participants noted they could turn their camera off and move in certain circumstances, such as large meetings and ‘in-house’ team meetings. Beyond online meetings, participants reported a perceived expectation to be visibly available online when w@h (e.g., through Microsoft 365 traffic light). This expectation manifested itself in a range of emotions outlined below in the automatic motivation theme.

Some participants reported that their colleagues were supportive and encouraged them to participate in activities or exercise. There were mixed findings about the supportive nature of organisational strategies to reduce SB. One participant highlighted that their line manager was supportive and understanding of the challenges of increased SB, but had a lack of knowledge about how to support, and was not able to offer suggestions on how to reduce their SB. Finally, participants commented how others in their household could have both a positive and negative impact on their SB. For example, some participants noted how a spouse could prompt them to break up their SB. Another participant highlighted that when their children returned from school it would result in further sitting.

##### Employee reflective and automatic motivation to reduce SB

As shown in Table 4, in relation to reflective motivation, participants shared their beliefs regarding the consequences of SB and reducing it. As noted, many participants were knowledgeable regarding the issue of SB, and this knowledge will inevitably also inform beliefs about the consequences of SB. In the theme belief about consequences of SB, comments relating specifically to participants own experiences of the consequences of SB were included. Participants reported that prolonged sitting negatively impacted their health, with Millie highlighting “*it was so surprising changing jobs from active. It’s, like, your muscles are sore but in a different way. Like, after exercising your muscles are sore but this time they’re, like, moaning….”* (Millie, 39, Transfusion Practitioner required to w@h). Others were aware that breaking up sitting would be beneficial for them. Additionally, some participants reported that they believed being physically active would compensate for their SB during the day.

**Table 4:** Themes relating to employee motivation to reduce SB when W@H

Category	General theme (and impact on reducing SB)	Sub-theme (where evident)
Reflective motivation	Beliefs about the consequences of SB (+)	Aware of negative consequences of prolonged SB
		Aware of the benefits of breaking up SB
		Believe PA can compensate for prolonged SB
	Beliefs about lack of capability to reduce SB (-)	Lack of time available
		Difficult
	Intention (or lack of) to move/reduce SB (+/-)	-
Automatic motivation	Emotional responses (+/-)	Anxious
		Feeling obligated
		Guilt
		Low mood
		Frustration with prompts
		Feeling the ‘need’ to move
		Positive emotions towards strategies to reduce SB

For those participants who reported believing they were not capable of reducing their SB, the majority reported concerns due to both lack of time, and that doing so would be ‘difficult’. Several participants highlighted that they intended to reduce their SB, for example one participant reported: *’My team already agreed we want to do, like, walking meetings’* (Freya, 32, Volunteer Co-ordinator). However, a number of participants highlighted that they either lacked intention or motivation to reduce their SB as illustrated by one participant who said: ‘*I’ve got a standing desk in the kitchen but it’s just making yourself do it I think is the problem*” (Harper, 37, Facilities Officer).

All themes within the category automatic motivation related to emotions experienced. As noted above in relation to social opportunity, participants reported a perceived expectation to be constantly visible and available online. This expectation resulted in feelings of anxiety, obligation, and guilt when not available. These responses were often due to concerns that others would judge that they were ‘skiving’. For example, one participant said: *‘….But I’ve got this real thing that if I’m in the kitchen and I don’t hear my email beep and I don’t immediately reply to that Teams message, that they might think I’m skiving*” (Emily, 37, Administrator). Experiencing low mood, and frustration with prompts from technology were additional emotions that may negatively impact reducing SB. Finally, some participants highlighted how they would experience a ‘need’ to move after prolonged SB, and one person reported feeling positive toward some of the ideas for reducing SB discussed during the focus group.

### Line managers

#### Line managers’ behaviour to support employees

Through the discussions, some line managers shared ways they supported their teams to reduce their SB, and these were clustered into three themes - managing workloads; encouraging breaks; and being considerate of team members’ working arrangements. Although managing workloads was not always explicitly about reducing SB, but rather about wider health and well-being implications, this theme was included because it could impact SB. Within this theme, line managers acknowledged they were aware of elevated work demands on employees because of w@h since the Covid-19 lockdown. Some had discussed this with their teams to emphasise the importance of focusing on outcomes and not presenteeism (i.e., being present but not productive), and encouraging employees to stick to contracted hours. For example, Sophia stated “… *I’ve been very adamant about sticking to your working hours, and not doing more to look after yourself, because I think that’s very important.*” (Sophia, 36, Head of Teaching Office). A number of line managers discussed how they explicitly encouraged employees to take breaks, and one discussed the importance of modelling this behaviour themselves. Finally, one participant noted that as a manager it was important to be considerate of the individual working arrangements of their employees.

#### Influences on line managers’ behaviour to support employees to reduce and/or break up SB when w@h

##### Line managers’ physical and psychological capability

As shown in Table 5, line managers’ comments about their capability to support their team to reduce SB when w@h included themes relating to psychological capability, but notably there were no comments on the impact of physical capability. Psychological capability related to knowledge about SB when w@h. A number of participants highlighted that they were knowledgeable about the ‘issue’ of SB including the elevated prevalence of SB generally and health consequences. Several participants noted that the information presented in the focus group infographic was not ‘surprising’, and many were also aware their own SB had increased. Participants had a good level of understanding about how most of the employees’ SB had elevated due to w@h; although this was not universal with some participants suggesting that there had not been a big change, and others indicating they had a lack of knowledge about employees SB. Many participants (although not all) had an understanding of what factors influenced an increase in SB, with many recognising the impact of changes in job demands, workload, ‘blurring’ of working days, and physical (home) environment. For example, one participant commented: “*They used to attend meetings across various bits of the campus, they used to go out to people and see them, wander round and have coffee and so on. So I think those figures are pretty accurate. But I do think 89 per cent is probably on the low side.”* (Archie, 48, Learning and Information Technology Manager). Similar to the employees, participants also recognised that working at home offered flexibility that increased opportunities to move during the working day, primarily through exercise.

**Table 5:** Themes relating to line managers psychological capability, physical and social opportunities and reflective motivation to support employees to reduce SB when w@h

Category	General theme (and impact on reducing SB)	Sub-theme (where evident)
Psychological capability	Knowledge about SB (+/-)	High level of knowledge about the SB ‘issue’
		Knowledge (or lack) of employees elevated SB when w@h
		Knowledge (or lack) of what is impacting employees SB when w@h
		Knowledge of increased opportunities to move
Physical opportunities	Organisational influences (+/-)	Activities (or lack of) for increased movement
		Having (or lack of) organisational policies and training
		Meeting etiquette (or lack of) (timing/scheduling)
	Nature of work (-)	Easy to interrupt via online/ add more demands
		Not suitable for moving meetings
	Access (or not) to equipment (+/-)	Equipment available (or not)
		Employees don’t have space to take up offer of equipment
	Reduced opportunities for employees to move (-)	In the home environment vs. office
		Due to work demands
		Loss of line managers-offered opportunities
Social opportunities	Social norms around online work (-)	Expectations on employees to be available online/at computer
		Social norm to be sitting
	Openly discuss SB/reduced movement (+)	-
	Supportive senior management (+)	-
Reflective motivation	Beliefs in benefits of supporting employees to reduce SB (+)	-
	Beliefs about low levels of capability to support employees to reduce SB (-)	Don’t effectively manage their own SB
		“Difficult”
	Intention to support employees to move/ reduce SB (+)	-

##### Line managers’ physical and social opportunities to support employees

The themes relating to physical opportunities represented the largest grouping and is presented in Table 5. Line managers highlighted that organisational influences such as providing opportunities to move more (e.g., set 5000 step goals) were helpful. It was also recognised that not having organisational policies and training for working at home, and specifically for online meeting etiquette was detrimental. Participants discussed the challenges of online meetings filling the whole day, back-to-back meetings, meetings being added to calendars by others, and meetings lasting one hour all of which reduced opportunities for movement. For example, Esme reported: “*there were issues in my institute where people were scheduling meetings at like eight o’clock in the morning and it’s just not on, and this was quite senior people, so there’s instructions going around about meeting etiquette and things like that, which I think do help.*” (Esme, 51, Business Lead).

Participants also noted that the nature of work had changed and it was easy to see if employees were online and available, and consequently they may be more likely to interrupt their employee’s work with an additional demand and thus keeping them at their desk. For example, Rory stated “*Whereas before that, you know, I wouldn’t have dreamt of contacting anyone during the day like that, I would have emailed at the end of the day, because I know most of them will be out on the road somewhere, or in meetings, or doing something else. So yeah, you get a little bit, a bit too familiar, if you like, you know, and you’re able to see where people are. And I don’t think it’s a good thing, necessarily, that you can sort of check on each other all the time, and feel that you can pull people in just whenever you want to talk to them*” (Rory, 45, Head of Innovation).

Line managers also noted the change in nature of work made it difficult to have moving meetings as screens were needed to share information, and notes had to be taken. Access (or not) to equipment (e.g., standing desks) was viewed as having an impact on being able to support employees. Mixed experiences were reported with some organisations having sufficient resources to provide equipment, and others did not. However, it was also recognised that some employees may not have space at home to install equipment like standing desks. Finally, the line managers recognised that the environment in which their employees were working impacted how they could support them, because employees had reduced opportunities to move when working at home compared with the office. These reduced opportunities were due to considerable work demands including high volume of online meetings and blurring of work-home boundaries, and there was a loss of line managers-led opportunities to move (e.g., organised exercise classes; walking groups).

There were limited comments from line managers relating to the role of social opportunities that may impact their behaviour in supporting employees to reduce SB, and these were clustered into four themes. The issue most commonly discussed related to the presence of an expectation that employees will be available online and at their computer. For example, Esme reported: “*I don’t know if some people feel that if they’re not glued to the front of their laptop or whatever device they’re using, are they deemed to not be working? So I think people feel a pressure to be seen to be active, you know?”* (Esme, 51, Business Lead). Some participants discussed how it is the social norm to sit when working or in meetings. Another participant commented that her team openly discussed the challenges of reduced movement creating an environment where this topic could be considered. Finally, one participant discussed how having an “*incredibly supportive*” chief executive enabled colleagues to purchase equipment to support w@h.

##### Line managers’ reflective and automatic motivation to support employees

There were limited comments relating to participants’ motivation to support their employees to reduce SB (see Table 5). Several participants highlighted that they believed encouraging their employees to move more would be beneficial for the employee. A couple of participants expressed concern that they would not be able to support their employees, either because they themselves did not manage their SB or because it would be ‘difficult’. Finally, some participants expressed that they had formed an intention to find ways to support their employees. There were no comments that clustered within automatic motivation.

### Employee and line managers perspectives on what may help them reduce their SB when w@h

Table 6 illustrates both employee and line managers’ suggestions on how employees could be supported to change behaviour and reduce SB. There were seven themes identified, with four.

**Table 6:** Employee and line managers’ suggestions on how to reduce SB when w@h

Suggestions to help reduce SB	Employee	Line managers
Moving whilst working	X	-
Behavioural regulation	X	-
Using equipment	X	X
Individual strategies	X	X
Organisational strategies	X	X
Culture shift	X	X
Provision of support by managers	-	X

common to both groups. Employees highlighted the potential of moving whilst working and included suggestions such as engaging in walking or standing meetings, or moving around the house whilst reading. Several employees discussed how they could use behavioural regulation strategies to manage their SB. Specifically, the potential of self-monitoring and prompts to trigger breaking up prolonged SB were identified. Additionally, several employees discussed how scheduled break times, managing work tasks, and making plans with others were important in ensuring they adhered to their plans. Employees also discussed the potential of equipment to support reducing SB, and specifically the use of standing desks and/or Swiss balls. The potential benefits of standing desks to support employees was also noted by several line managers, although it was also recognised that this was not always possible due to resources nor employees having suitable space.

A further employee theme related to individual strategies to encourage movement such as ensuring they got dressed and wore shoes, got a dog, or played music. Only one comment from line managers related to individual strategies and this was a suggestion that employees should block out time in their calendar for movement. Both employees and line managers flagged the importance of organisational initiatives in supporting employees to be able to reduce SB and move more. A number of both employees and line managers highlighted the value of organised challenges (e.g., Step Count Challenge). Others from both groups highlighted how the implementation of organisation-wide initiatives such as being assigned time within the working day to be active, and managing scheduling of meetings to encourage breaks and movement (e.g., 50 min meetings/ no meeting Fridays) would be valuable and help support a culture shift. However, one participant flagged the challenges of implementing organisational initiatives as they may not be fully inclusive. Further, both employees and line managers noted the need for a culture shift and the importance of flexible working and activities such as moving meetings being normalised and perceived as ‘acceptable’. The employees suggested that managers should be ‘leading the way’ with a culture shift. Linked to this point, a number of line managers discussed how as managers they could provide support to help employees move more. This support could be through encouraging teams to take breaks and move during meetings. Additionally, one participant indicated that it was beneficial to acknowledge worker’s feelings of guilt around moving away from the desk.

Based on the findings of our rapid review [14], both employees and line managers were asked to consider if and how the identified strategies could work in their w@h environment. There were mixed opinions from employees and line managers regarding prompts, with some indicating that they do not work, and many highlighting that on their own prompts will not work. Participants suggested various ways to enhance the use of prompts including individualising them, having them come from the organisation (instead of set by self), having others use prompts too, being held accountable for responding to prompts, and having an incentive to respond to a prompt. With regards to education, this was viewed as a valuable strategy by employees to raise knowledge, and would be most valued if it could be individualised and should also be targeted at management levels. Line managers also had suggestions on how to optimise education that included ‘drip-feeding’ information regularly, focusing on the most powerful information (e.g., using data on sedentary levels), providing education on a voluntary basis, delivering by ‘stealth’ in settings where the focus is on another topic, and incorporating with other strategies such as prompts and feedback. Feedback was viewed as valuable by both groups, and particularly if it was also individualised and one participant highlighted the importance of aligning feedback with lifestyle goals (e.g., achieve 10,000 steps). One line manager flagged that they did not think feedback would be well received as in general employees know what they should be doing. Participants were positive regarding workplace initiatives, with line managers noting that they were valued in part due to them signalling to employees that the organisation validated breaks and movement. Further, one line manager highlighted the challenge of getting all team members to engage in workplace initiatives, and another flagged the importance of ensuring initiatives are balanced against the organisational needs. Only one line manager commented on supportive work environment, and highlighted the importance of support coming from the ‘top’ of the organisation. There was only one comment, from an employee, on the potential of training with a suggestion to keep it simple.

## Discussion

Since Covid-19, the prevalence of w@h has increased dramatically and created a high-risk setting for elevated SB [10, 11]. This is the first study to consult with both employees and line managers in the w@h environment to better understand the target behaviour, what needs to change, and identify potential strategies to support employees aged 18–40 to reduce and/or break up SB. The findings of this study have the potential to provide an original and valuable contribution to the growing evidence to inform intervention development in this context.

Most employees and line managers perceived that employee’s SB was elevated in the w@h environment, and this is consistent with review evidence relating to changes in workers SB during Covid-19 [10], and observed increases in SB when w@h compared with the office [24]. Nevertheless, some participants highlighted that w@h provided them with opportunity to move during the day and compensate for SB by being active at other times of the day, suggesting a complex picture. There is a need to better understand how w@h impacts SB and related movement behaviours.

Using the COM-B theoretical lens [25], it was clear that there were multiple influences on employee’s SB; although some factors were discussed more fully than others, suggesting they may be more influential. For example, there were few comments from these participants relating to physical capability, and this is consistent with previous research at home [11] and in the office [26–28] suggesting this is not a major influence on occupational SB. Nevertheless, it is important to acknowledge that there will be sectors of the population who do have physical limitations to reducing SB, and strategies and language should be inclusive [29].

Within psychological capability, knowledge about SB levels and its health consequences was discussed. Although these participants were generally a well-informed group, it was evident that the more nuanced distinction between SB and PA was not always fully understood with several participants indicating that they compensated for high levels of SB with exercise. Current health recommendations emphasise the importance of *both* minimising SB and being PA for optimal health [30], and this message needs to be highlighted through careful education. Although the participants did not explicitly highlight education as a feasible strategy, both employees and line managers were receptive to educational materials as a suggestion that had been identified as promising from our rapid review [14].

Physical opportunities was one of the largest themes, illustrating the substantial impact of the environmental change from the move to w@h. Employees identified a number of factors that had reduced their opportunity to move during the w@h day including, for example, loss of commute and increased work demands that resulted in increased, uninterrupted desk time. This finding is consistent with our previous research [11] where physical opportunities to reduce sitting were perceived to be limited in the w@h environment, but influential on behaviour. Office-based research has also noted the negative impact of physical opportunities and work demands on SB behaviour [26, 27, 31]. A finding unique to the w@h environment was the negative impact of the blurring of boundaries between work and home, where participants struggled to (literally) step away from work. Other research has noted the challenge of separating personal and work life [32, 33], and these findings highlight the additional impact on SB when w@h. It is noteworthy that these experiences were not universal and encouragingly, some participants acknowledged that w@h provided increased opportunities for movement as it provided flexibility, they recouped time from no longer commuting, or were able to engage in non-seated meetings. Providing workers with educational materials and examples of opportunities to move more afforded by the w@h environment may be a useful intervention strategy, and is supported by findings from the rapid review [14]. For example, participants themselves identified such strategies including active meetings, using equipment, and planning and scheduling opportunities to move. Additionally, previous research has shown that introducing standing desks at home can be beneficial [15], although participants in this current study did acknowledge not all employers nor employees had the resources and/or space for these.

The social opportunity theme was not large relative to others but included themes unique to the nature of the w@h environment. Employees reported social norms around online meetings that generally did not encourage movement. Further, several participants reported that they perceived an expectation to be visible and available online that restricted their capacity to move away from the desk. These findings reinforce our previous research [11] where participants reported low levels of social opportunity to reduce sitting when w@h, and it was suggested that the digital environment can create space where participants feel observed and monitored. Further, office-based studies have also reported that social norms, and expectations of others can negatively impact sitting behaviour [26, 27, 31]. As suggested by participants, there is a role for organisations in implementing a culture shift to challenge social norms and make strategies to reduce SB more acceptable with managers actively and visibly leading the way, and implementing organisation-wide initiatives. This finding complements the findings of the rapid review that highlighted the promise of using role models to target behaviour change [14].

Employees had generally positive beliefs about the consequences of reducing SB, and contrary to previous research [11, 26] there was limited concern in this sample regarding the impact of reducing SB on productivity. Some participants reported that they believed they lacked capability to reduce SB, and building self-efficacy in this behaviour may be important to facilitate behaviour change. With regards to automatic motivation, consistent with our previous research there were few comments relating to the habitual nature of the behaviour [11]. However, a number of participants reported negative emotive responses linked to the social environment that could impact behaviour. For example, feelings of guilt, anxiety and obligation were associated with the perceived need to be visible, and may consequently reduce the likelihood of reducing SB. As noted above, cultural change is needed to address such issues, challenge norms and have open discussions around what is acceptable/ healthy would be beneficial.

It was clear from the employees’ responses and other research [11, 26–28] that the social environment and the actions of managers and the wider organisation could set the tone for encouraging greater movement during the day. These findings reinforce the importance of understanding the line managers’ perspectives in supporting W@H, and this study makes an original contribution to that understanding. Line managers discussed a number of behaviours that they engaged in to support their employees to reduce SB. Line managers’ main focus was around the importance of supporting employees to manage their workload, avoid presenteeism and stick to working hours, all of which could help address workload as a perceived barrier to employees’ behaviour. Additionally, line managers discussed explicitly encouraging and modelling taking breaks, which is consistent with employees’ suggestions that managers should actively and visibly support SB reduction.

Using the COM-B theoretical lens [25], is also useful to understand what can impact line managers’ ability to support employees to inform the design of feasible interventions. There were no comments relating to physical capability and all comments relating to psychological capability related to knowledge about SB. Many line managers, although not all, had a good level of understanding regarding the issue of SB and relationship with health, and most knew that SB had been elevated in employees w@h. Consistent with employees’ responses, most line managers had an awareness that SB had been impacted by job demands, the blurring of the workday, and the impact of the home environment. These findings are encouraging suggesting that, for these line managers, there is limited need to educate and raise awareness of the issue.

Line managers discussed how the physical opportunities of the w@h environment impacted how they could support employees, and there were similarities with the employees’ comments. For example, line managers recognised that there were reduced opportunities for employees to move at home, and that availability of equipment could help. Also consistent with employees, there was recognition of the role of the organisation and specifically how having (or not having) activities to encourage movement, policies and training on the issue, and etiquette around length and scheduling of meetings could all be influential. Strategies at an organisational level were identified and included engaging with external workplace initiatives, scheduling meetings to encourage movement, and having specific time for movement.

In terms of social opportunity, like employees, line managers also recognised the impact of social norms around sitting, and expectations to be online and available that pervaded the work environment. Having an environment where the topic was discussed and senior managers were supportive was viewed as important. Line managers noted that acknowledging emotions around not being at one’s desk could be helpful, and this is encouraging as it is clear that employees experience a range of negative emotions regarding moving from their desk. Similar to employees, line managers also recognised the need for a cultural shift to normalise more movement and challenge current social norms. With regards to motivation, line managers generally recognised the benefits of supporting employees to reduce SB, but some were not confident in their ability to do so.

### Future research

In line with the MRC framework [13], the purpose of this study was to inform future intervention development. It was clear that there are multiple influences on SB when w@h, and no single intervention strategy will be beneficial for all. Informed by the findings from this study and the programme of research to date [11, 14], it is recommended that future research should design, implement and evaluate a toolkit of resources to address individual needs, and support effective behaviour change. Consistent with theoretical perspectives on the process of behaviour change [34], for some employees and line managers there will be a need to provide educational resources on levels and consequences of SB in the w@h environment, and the distinction between SB and physical activity, as well as the need to meet health recommendations for both SB and physical activity. Increased knowledge will be needed in order to support intention formation to change behaviour. Individuals who already intend to reduce their SB may benefit from other elements of the toolkit. It will be challenging to modify the w@h environment for all, so education and instructions on how to reduce SB when w@h will be critical. Behavioural regulation strategies are also needed to facilitate behaviour change, and within a toolkit, prompts to move and tools to support action planning are examples. It has been suggested that making a plan to be prompted to move at the point of progressing from one task to another (i.e., at task boundaries), may be more effective than time-based prompts or schedules [35]. It was starkly evident in the findings that the social environment is highly influential, and both groups highlighted the need for cultural change to normalise moving during the w@h day. Cultural change is challenging, and emphasises the need for senior management buy-in and the implementation of organisational initiatives to support change [36]. The toolkit could include initiatives that could be integrated into existing organisational infrastructure to support wide-spread change (e.g., apps for MS Office 365). Furthermore, at an individual level, providing support to line managers to be able to role model behaviour change, and proactively provide social support would be beneficial. A toolkit of resources should be developed, but any adaptations and implementation must be undertaken in consultation with the target organisations [13]. An additional research priority is to develop a better understanding of how w@h for some or all of the working week impacts SB and related movement behaviours. In line with contemporary perspective, future research should use device-based assessment of SB, physical activity and sleep across a 24-hour period [37] in the w@h environment.

### Strengths

This study has several strengths that ensured the study made a robust contribution to the evidence base. Firstly, the study is part of a programme of research informed by contemporary guidance on intervention development and evaluation, and integrated a strong theoretical framework. As part of this programme of research, the integration of the rapid review findings [14] into the focus group format ensured the discussion with the participants was centred on the most up to date understanding. A further unique strength is the inclusion of both employees from a broad range of desk-based roles and line managers in order to gain a fuller understanding on influences on and potential strategies to reduce and break up SB when w@h.

### Limitations

It is likely that the sample may have been biased, as those who volunteered to participate in the study were likely to have an interest in the topic and an awareness of the issue. Further, providing information on the topic prior to and at the start of the focus group in order to raise awareness of the behaviour [22] may have impacted on the participants’ responses. Additionally, participants were predominantly female and white, and did not indicate any physical limitations that could impact their capability to reduce and break up SB. It is important to acknowledge that these findings may not be representative of the broader w@h population, and future research should aim to recruit more representative samples.

## Conclusion

The working landscape has transformed, and there is an urgent need to support the health and well-being of employees who are w@h. The findings of this study make a potentially important and original contribution to our understanding of the nature of and influences on SB when w@h, from both employee and line managers perspectives. It is clear that there are multiple influences on SB when w@h, and behaviour change interventions should incorporate a range of strategies at both individual and organisational levels. Future intervention research is a priority to evaluate and refine strategies and inform w@h guidance to mitigate both the short-term and long-term health consequences of elevated SB.

### Electronic supplementary material

Below is the link to the electronic supplementary material.

Supplementary Material 1

Supplementary Material 2

Supplementary Material 3

Supplementary Material 4

## Data Availability

The datasets generated in this study are available https://datashare.ed.ac.uk/handle/10283/8473 and are de-identified, with pseudonyms included. Where necessary, information that could potentially be identifiable has been redacted. Supplementary file 4 provides an example of the focus group materials, but the full materials are available https://osf.io/z7tvy/.

## Abbreviations

SB: sedentary behaviour
w@h: work at home

## References

[1] Tremblay MS, Aubert S, Barnes JD, Saunders TJ, Carson V, Latimer-Cheung AE (2017). Sedentary Behavior Research Network (SBRN) - terminology Consensus Project process and outcome. Int J Behav Nutr Phys Act.

[2] 2018 Physical Activity Guidelines Advisory Committee. 2018 Physical Activity Guidelines Advisory Committee Scientific Report,. Washington D.C.; 2018 2018.

[3] Del Alfonso-Rosa PCB, McGregor RM, Chastin D, Palarea-Albaladejo SF, Del Pozo Cruz J (2020). Sedentary behaviour is associated with depression symptoms: compositional data analysis from a representative sample of 3233 US adults and older adults assessed with accelerometers. J Affect Disord.

[4] Allen MS, Walter EE, Swann C (2019). Sedentary behaviour and risk of anxiety: a systematic review and meta-analysis. J Affect Disord.

[5] Dzakpasu FQS, Carver A, Brakenridge CJ, Cicuttini F, Urquhart DM, Owen N (2021). Musculoskeletal pain and sedentary behaviour in occupational and non-occupational settings: a systematic review with meta-analysis. Int J Behav Nutr Phys Activity.

[6] UK Chief Medical Officers. UK Physical Activity Guidelines. 2019.

[7] Bull FC, Al-Ansari SS, Biddle S, Borodulin K, Buman MP, Cardon G (2020). World Health Organization 2020 guidelines on physical activity and sedentary behaviour. Br J Sports Med.

[8] Parry S, Straker L (2013). The contribution of office work to sedentary behaviour associated risk. BMC Public Health.

[9] Office for National Statistics. Working from home in NUTS1 regions. https://www.ons.gov.uk/employmentandlabourmarket/peopleinwork/employmentandemployeetypes/adhocs/12570workingfromhomeinnuts1regions; 2020.

[10] Ráthonyi G, Kósa K, Bács Z, Ráthonyi-Ódor K, Füzesi I, Lengyel P (2021). Changes in workers’ physical activity and sedentary behavior during the COVID-19 pandemic. Sustainability.

[11] Niven A, Baker G, Almeida EC, Fawkner SG, Jepson R, Manner J (2023). Are we working (too) comfortably? Understanding the nature of and Factors Associated with sedentary Behaviour when working in the Home Environment. Occup Health Sci.

[12] Office for National Statistics. Characteristics of homeworkers, Great Britain: September 2022 to January 2023, 2023 [ https://www.ons.gov.uk/employmentandlabourmarket/peopleinwork/employmentandemployeetypes/articles/characteristicsofhomeworkersgreatbritain/september2022tojanuary2023#:~:text=Overall%2C%2044%25%20of%20workers%20reported,reporting%20working%20from%20home%20only

[13] Skivington K, Matthews L, Simpson SA, Craig P, Baird J, Blazeby JM (2021). A new framework for developing and evaluating complex interventions: update of Medical Research Council guidance. BMJ.

[14] Morton S, Fitzsimons C, Jepson R, Saunders DH, Sivaramakrishnan D, Niven A. What works to reduce sedentary behavior in the office, and could these intervention components transfer to the home working environment? A rapid review and transferability appraisal. Front Sports Act Living. 2022;4. 10.3389/fspor.2022.954639 PMC9372484 35966113

[15] Falk GE, Mailey EL, Okut H, Rosenkranz SK, Rosenkranz RR, Montney JL et al. Effects of Sedentary Behavior interventions on Mental Well-Being and Work Performance while Working from Home during the COVID-19 pandemic: a pilot randomized controlled trial. Int J Environ Res Public Health. 2022;19(11). 10.3390/ijerph19116401 PMC9180109 35681986

[16] Mailey EL, Rosenkranz R, Rosenkranz SK, Ablah E, Talley M, Biggins A (2022). Reducing Occupational sitting while Working from Home: Individual and Combined effects of a height-adjustable desk and an online behavioral intervention. J Occup Environ Med.

[17] Michie S, Atkins L, West R. The Behaviour Change Wheel: a guide to designing interventions. Silverback Publishing; 2014.

[18] Keyworth C, Epton T, Goldthorpe J, Calam R, Armitage CJ (2020). Acceptability, reliability, and validity of a brief measure of capabilities, opportunities, and motivations (COM-B). Br J Health Psychol.

[19] Strain T, Kelly P, Mutrie N, Fitzsimons C (2018). Differences by age and sex in the sedentary time of adults in Scotland. J Sports Sci.

[20] Kazi A, Haslam C, Duncan M, Clemes S, Twumasi R (2019). Sedentary behaviour and health at work: an investigation of industrial sector, job role, gender and geographical differences. Ergonomics.

[21] Malterud K, Siersma VD, Guassora AD (2016). Sample size in qualitative interview studies:guided by Information Power. Qual Health Res.

[22] Gardner B, Flint S, Rebar AL, Dewitt S, Quail SK, Whall H (2019). Is sitting invisible? Exploring how people mentally represent sitting. Int J Behav Nutr Phys Activity.

[23] Braun V, Clarke V (2022). Thematic analysis: a practical guide.

[24] Olsen HM, Brown WJ, Kolbe-Alexander T, Burton NW (2018). Flexible work: the impact of a New Policy on employees’ sedentary behavior and physical activity. J Occup Environ Med.

[25] Michie S, Atkins L, West R (2014). The Behaviour Change Wheel: a Guide to Designing interventions.

[26] Macdonald BW, Fitzsimons C, Niven A, editors. Using the COM-B model of behaviour to understand sitting behaviour in U.K. office workers2018.

[27] Munir F, Biddle SJH, Davies MJ, Dunstan D, Esliger D, Gray LJ (2018). Stand more AT work (SMArT work): using the behaviour change wheel to develop an intervention to reduce sitting time in the workplace. BMC Public Health.

[28] Ojo SO, Bailey DP, Hewson DJ, Chater AM. Perceived barriers and facilitators to breaking up sitting time among desk-based Office workers: a qualitative investigation using the TDF and COM-B. Int J Environ Res Public Health. 2019;16(16). 10.3390/ijerph16162903 PMC6720704 31416112

[29] Smith B, Mallick K, Monforte J, Foster C (2021). Disability, the communication of physical activity and sedentary behaviour, and ableism: a call for inclusive messages. Br J Sports Med.

[30] World Health Organization. WHO guidelines on physical activity and sedentary behaviour. https://apps.who.int/iris/rest/bitstreams/1315866/retrieve; 2020. 33369898

[31] Ojo SO, Bailey DP, Brierley ML, Hewson DJ, Chater AM (2019). Breaking barriers: using the behavior change wheel to develop a tailored intervention to overcome workplace inhibitors to breaking up sitting time. BMC Public Health.

[32] The Scottish Government. Working from home during the COVID-19 pandemic: benefits, challenges and considerations for future ways of working. 2022.

[33] Keightley S, Duncan M, Gardner B (2023). Working from home: experiences of Home-Working, Health Behavior and Well-being during the 2020 UK COVID-19 Lockdown. J Occup Environ Med.

[34] Schwarzer R, Hamilton K, Hamilton K, Cameron LD, Hagger MS, Hankonen N, Lintunen T (2020). Changing Behavior using the health action process Approach. The handbook of Behavior Change. Cambridge Handbooks in psychology.

[35] ten Broeke P, Gardner B, Beckers DGJ, Geurts SAE, Bijleveld E. Why do people sit? A framework for targeted behavior change. Health Psychology Review.1–14. 10.1080/17437199.2022.2143851 36343923

[36] Jepson R, Baker G, Sivaramakrishnan D, Manner J, Parker R, Lloyd S et al. Feasibility of a theory-based intervention to reduce sedentary behaviour among contact centre staff: the SUH stepped-wedge cluster RCT.10:13. 36538605

[37] Rollo S, Antsygina O, Tremblay MS (2020). The whole day matters: understanding 24-hour movement guideline adherence and relationships with health indicators across the lifespan. J Sport Health Sci.

