# Supplementary material for: “Are we working (too) comfortably?”: a focus group study to understand sedentary behaviour when working at home and identify intervention strategies
Source: BMC Public Health. 2024 Jun 6;24:1516. doi: 10.1186/s12889-024-18892-1 (PMC11155077; doi:10.1186/s12889-024-18892-1)
Supplement: Supplementary file 2 — Supplementary Material 2 [file 12889_2024_18892_MOESM2_ESM.docx]

Supplementary file 2: Participant flow through recruitment to focus group

Participated in employee focus group (n=21)

Dropped out during FG (n=1)

Ineligible (n=1)

Failed to select FG (n=2)

Waiting list (n=5)

Unavailable (n=2)

If meet inclusion criteria, invited to complete consent form

Expression of interest received (n=92)

Participants received study information

Invited to select one of three employee focus groups (n=32)

Unavailable (n=1)

Failed to select FG (n=5)

Waiting list (n=1)

Invited to select one of three line manager focus groups (n=28)

Participated in employee focus group (n=21)

Recruitment open (Dec 2021- Feb 2022)^1^

^1^ University of Edinburgh staff e-noticeboards (incl. MS Teams posts), social media (Twitter), and through a blog hosted on a stakeholder’s website ([Are you working too comfortably? (stepcount.org.uk)](https://www.stepcount.org.uk/blog/are-you-working-too-comfortably))
