# Supplementary material for: “Are we working (too) comfortably?”: a focus group study to understand sedentary behaviour when working at home and identify intervention strategies
Source: BMC Public Health. 2024 Jun 6;24:1516. doi: 10.1186/s12889-024-18892-1 (PMC11155077; doi:10.1186/s12889-024-18892-1)
Supplement: Supplementary file 3 — Supplementary Material 3 [file 12889_2024_18892_MOESM3_ESM.pdf]

THE UNIVERSITY of EDINBURGH
Moray House School of Education and Sport

Break it Up!

Reducing long periods of desk time whilst working from home

Focus Group – with employees

Jan / Feb 2022

UKRI Medical Research Council

RECORD

THE UNIVERSITY of EDINBURGH
Moray House School of Education and Sport

Meeting Agenda

- Introductions and housekeeping
- Project overview / aims
- Over to you - discussions and ideas for breaking up sitting while working at home
- Solutions that work in the office – what do you think?!
- Next steps

UKRI Medical Research Council

Break it Up!

THE UNIVERSITY of EDINBURGH
Moray House School of Education and Sport

Flash intros!

- Name
- Job role
- Where you work
- Tea or coffee?!
- Favourite biscuit?

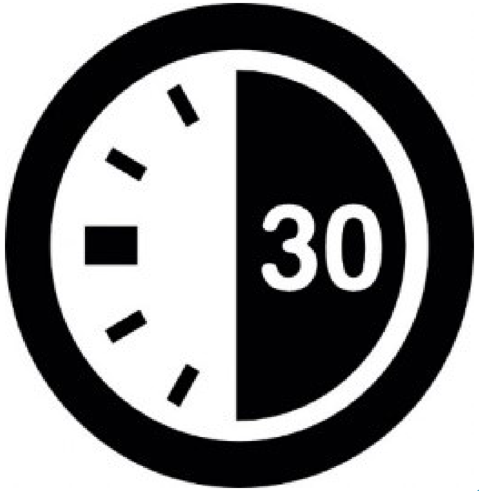

Housekeeping

THE UNIVERSITY *of* EDINBURGH
Moray House School of Education and Sport

- **No right and wrong answers.** All opinions are valuable.
- **Only one person speaking at a time** – it helps with transcribing!
- We're **recording the discussion** - what you say is **confidential** and stays in this room. We won't identify you in our reports / publications.
- Please **do not share discussions** outside this room.
- Please **be respectful** of everyone's opinions.
- You are **free to leave** at any time without giving a reason.
- Feel free to **use the chat** function for any links / suggestions / points / etc.
- And of course – **stand up / stretch / move anytime you wish**

UKRI Medical Research Council

Break it Up!

COVID lockdown has led us to **new ways of working**

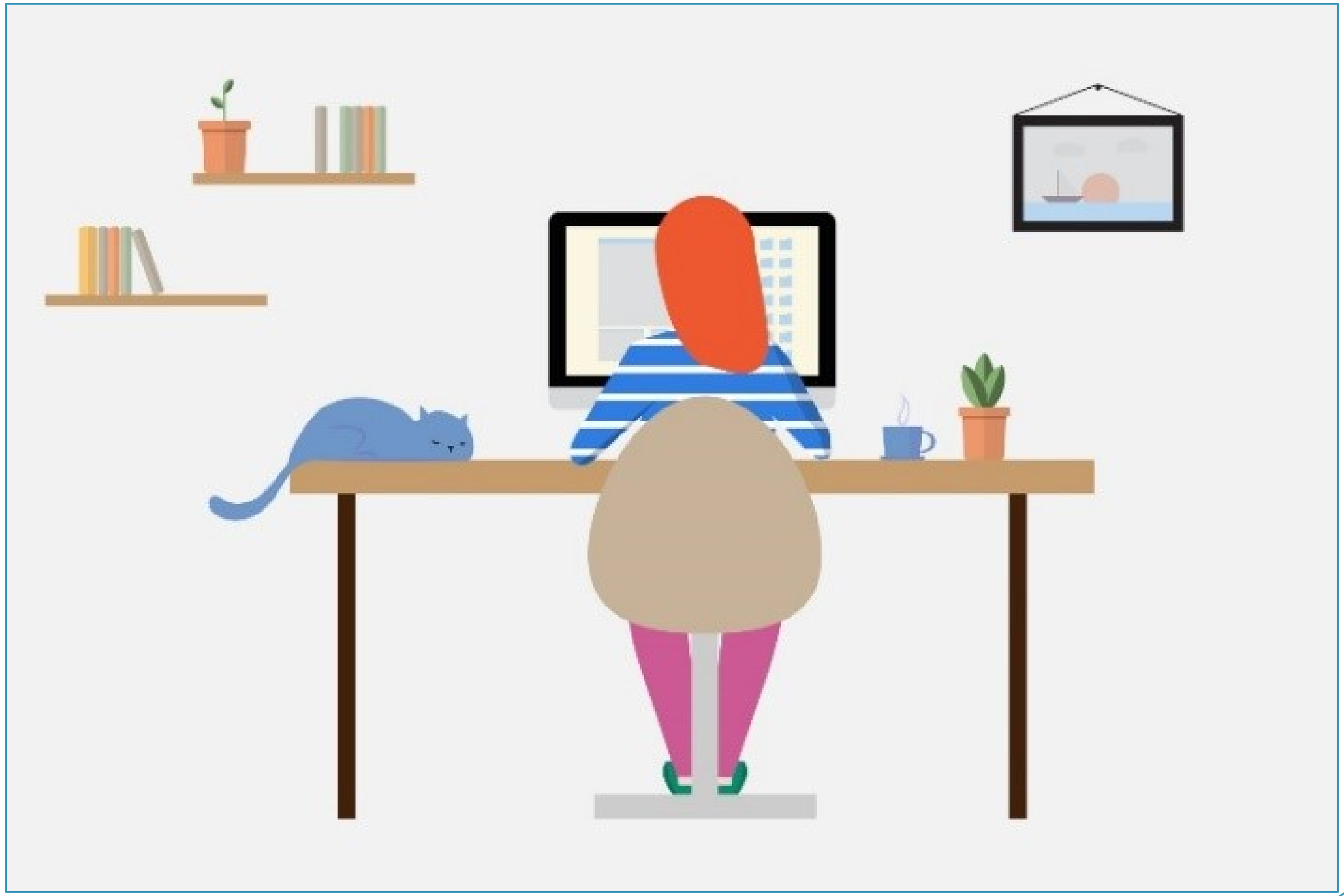

© Emilija Randjelovic via Getty Images

THE UNIVERSITY of EDINBURGH
Moray House School of Education and Sport

UKRI Medical Research Council

Break it Up!

We need **<u>your input</u>** to:

1. Understand challenges while working from home
2. Identify where there are opportunities to break up sitting while working at home

WHAT ARE THE RISKS OF TOO MUCH SITTING?

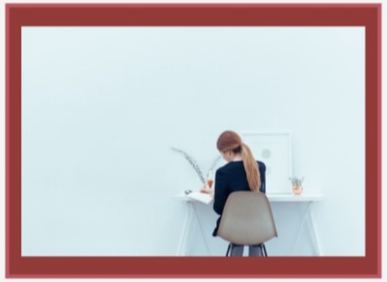

- High levels are associated with:
  - increased risk of all-cause cardiovascular disease and cancer mortality
  - increased likelihood of developing cardiovascular disease, some cancers, or type 2 diabetes
- Also associated with negative impact on mental health:
  - increased risk of anxiety, depression, and sleep disorders
  - lower levels of emotional wellbeing
- Risk can be reduced by moving throughout the day. [1]

HOW HAS WORKING FROM HOME CHANGED OFFICE WORKERS SITTING BEHAVIOUR?

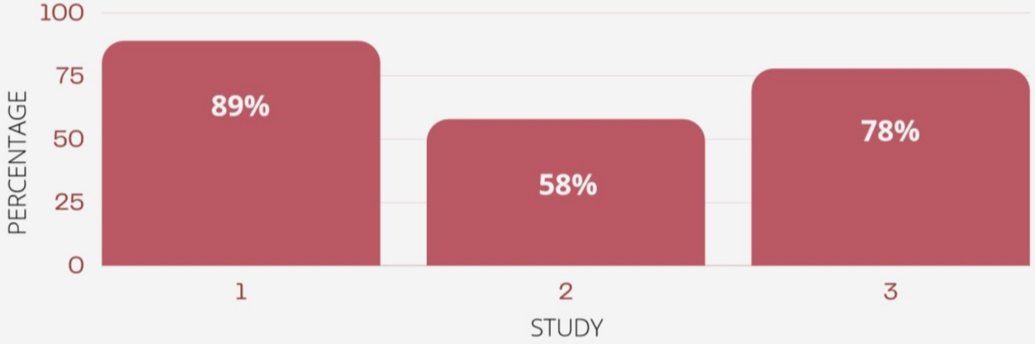

- Study 1 – Working at home (Niven et al, in prep) [2]
- Study 2 – Office based workers (Maes et al, 2020) [3]
- Study 3 – Office based workers (Rosenkranz et al, 2020) [4]

All data from self reported OSPAQ questionnaire [5]

THE UNIVERSITY of EDINBURGH
Moray House School of Education and Sport

UKRI Medical Research Council

Break it Up!

NOW OVER TO YOU!

How have you found working from home?

- **<u>What did you think of the information on the infographic about sitting?</u>**
- Are you sitting more / less than you were when you were working in the office? What influences this?
- Is there anything you can think of that would be a good idea for breaking up sitting – even if you haven't already tried it out yourself?

THE UNIVERSITY of EDINBURGH
Moray House School of Education and Sport

NOW OVER TO YOU!

How have you found working from home?

- What did you think of the information on the infographic about sitting?
- **Are you sitting more / less than you were when you were working in the office? What influences this?**
- Is there anything you can think of that would be a good idea for breaking up sitting – even if you haven't already tried it out yourself?

THE UNIVERSITY of EDINBURGH
Moray House School of Education and Sport

UKRI Medical Research Council

Break it Up!

NOW OVER TO YOU!

How have you found working from home?

- What did you think of the information on the infographic about sitting?
- Are you sitting more / less than you were when you were working in the office? What influences this?
- **Is there anything you can think of that would be a good idea for breaking up sitting - even if you haven't already tried it out yourself?**

THE UNIVERSITY of EDINBURGH
Moray House School of Education and Sport

Medical Research Council

Break it Up!

These are some ideas we have identified that **work in the office**

How do you think they would work at home?

Education about SB and the health consequences

Regular **prompts**

Personal and/or individual **feedback** on SB

THE UNIVERSITY of EDINBURGH
Moray House School of Education and Sport

UKRI Medical Research Council

Next steps following todays discussions

- Next steps:
  - More focus groups – with line managers and employees (during Jan and Feb 2021)
  - **Workshops** – to review findings from the focus groups and present our preliminary solutions (planned for spring)
  - **Thank you voucher** – Sarah will be in touch soon to arrange
  - We will **keep in touch with updates** about the project – unless you ask us not to

THE UNIVERSITY of EDINBURGH
Moray House School of Education and Sport

UKRI Medical Research Council

THANK YOU
FOR YOUR TIME AND INPUT TODAY

We are still recruiting participants for our focus groups - if you are able to share details about our project with your networks – please get in touch.

Ailsa.niven@ed.ac.uk; sarah.morton@ed.ac.uk; claire.fitzsimons@ed.ac.uk; Divya.Sivaramakrishnan@ed.ac.uk

@ailsaniven @_sarah_morton @CFFitzsimons @DivyaSivaramak

THE UNIVERSITY of EDINBURGH Moray House School of Education and Sport

UKRI Medical Research Council

Break it Up!
