# Supplementary material for: “Are we working (too) comfortably?”: a focus group study to understand sedentary behaviour when working at home and identify intervention strategies
Source: BMC Public Health. 2024 Jun 6;24:1516. doi: 10.1186/s12889-024-18892-1 (PMC11155077; doi:10.1186/s12889-024-18892-1)
Supplement: Supplementary file 4 — Supplementary Material 4 [file 12889_2024_18892_MOESM4_ESM.pdf]

THE UNIVERSITY of EDINBURGH
Moray House School of Education and Sport

Break it Up!
Reducing long periods of desk time whilst working from home

Are we working too comfortably?

Developing an intervention to reduce sedentary behaviour and prolonged sitting while working from home

Research team: Dr Ailsa Niven, Dr Claire Fitzsimons, Dr Sarah Morton, Dr Divya Sivaramakrishnan, Dr Dave Saunders, Dr Ruth Jepson
Physical Activity for Health Research Centre, University of Edinburgh

A 'NEW' NORMAL

Covid has led us to new ways of working, with large proportions of the population now spending much of their working week in the home environment. While there are many positives, there are also some negatives - one of which is that we may be spending more time in prolonged seated postures which could lead to a range of short and long term physical and metal health issues. There is a need to support those working from home to in a safe and healthy way. Our project focuses on developing an intervention to reduce sitting time while working from home.

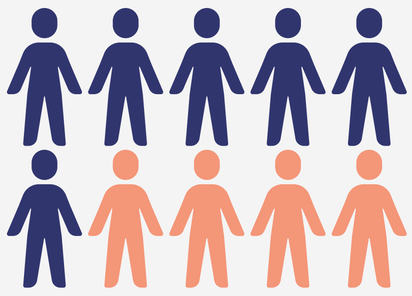

WHAT ARE THE RISKS OF TOO MUCH SITTING?

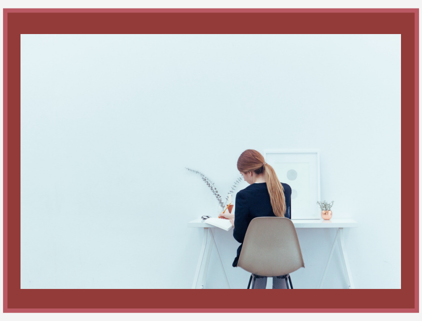

- High levels are associated with:
  - increased risk of all-cause CVD and cancer mortality
  - increased CVD, cancer, and type 2 diabetes incidence
- Also associated with negative impact on mental health:
  - increased risk of anxiety, depression, and sleep disorders
  - lower levels of emotional wellbeing
- Risk can be mitigated through changes in: (1) physical activity, (2) sedentary time, (3) both (ideal situation). [1]

HOW HAS WORKING FROM HOME CHANGED OFFICE WORKERS SITTING BEHAVIOUR?

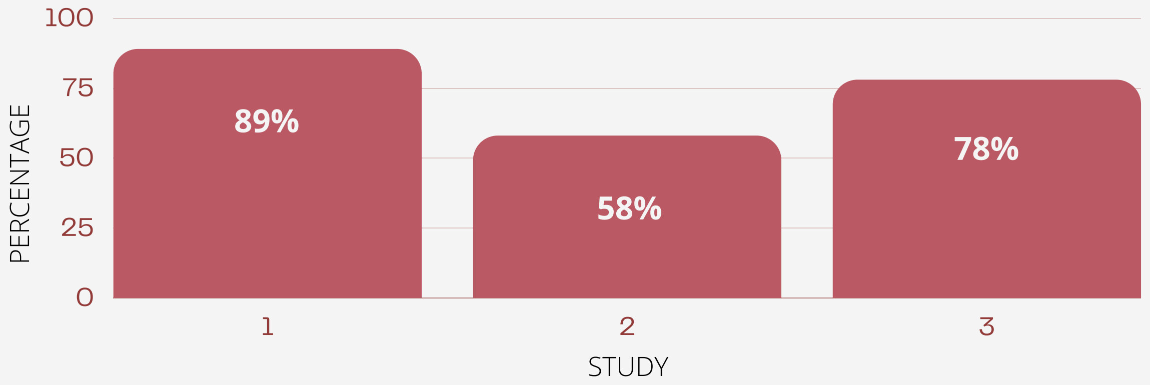

- Study 1 - Working at home (Niven et al, in prep) [2]
- Study 2 - Office based workers (Maes et al, 2020) [3]
- Study 3 - Office based workers (Rosenkranz et al, 2020) [4]

All data from self reported OSPAQ questionnaire [5]

OUR APPROACH TO DEVELOPING A SOLUTION

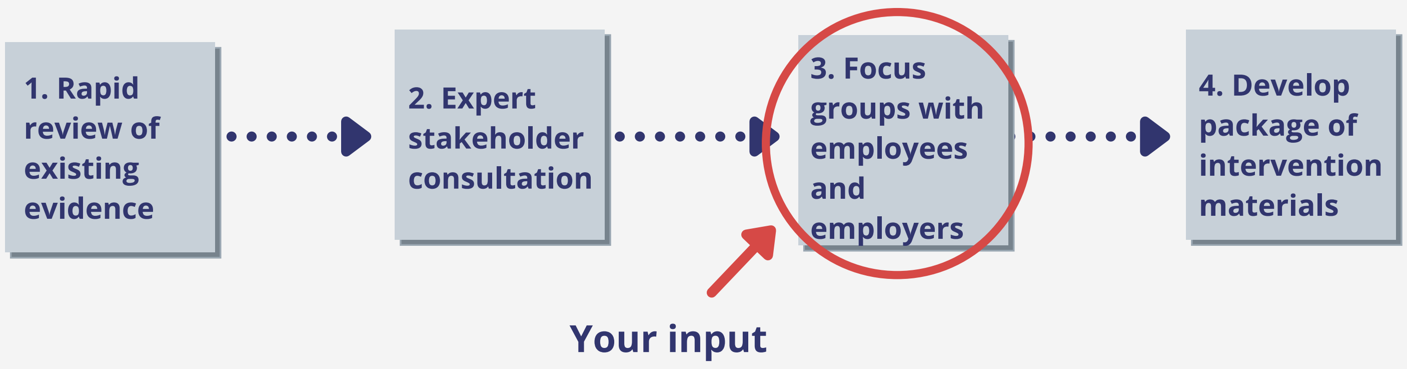

WHAT CAN WE DO TO SUPPORT THOSE WHO ARE WORKING FROM HOME TO MOVE MORE?

This is where you come in!

At the focus group, we would like to hear from you about your experiences:
- are you sitting more or less than you did in the office?
- what influences the time you spend sitting while working at home?
- have you come up with any interesting / innovative ways to move more?

We'll also share some of our ideas - and we'd be keen to hear what you think of them.

References

[1] Dempsey et al., New global guidelines on sedentary behaviour and health for adults: broadening the behavioural targets. IJBNPA, November 2020
US 2018 Physical Activity Guidelines Advisory Committee Scientific Report. Washington, DC: U.S Department of Health and Human Services. 2018
[2] Niven et al, in prep
[3] Maes, I., Ketels, M., Van Dyck, D. and Clays, E., 2020. The occupational sitting and physical activity questionnaire (OSPAQ): a validation study with accelerometer-assessed measures. BMC public health, 20(1), pp.1-10.
[4] Rosenkranz, S.K., Mailey, E.L., Umansky, E., Rosenkranz, R.R. and Ablah, E., 2020. Workplace sedentary behavior and productivity: a cross-sectional study. International Journal of Environmental Research and Public Health, 17(18), p.6535.
[5] Microsoft Word - Appendix OSPAQ items.doc (lww.com)
